# Effects of low intensity pulsed ultrasound on expression of B-cell lymphoma-2 and BCL2-Associated X in premature ovarian failure mice induced by 4-vinylcyclohexene diepoxide

**DOI:** 10.1186/s12958-021-00799-w

**Published:** 2021-07-20

**Authors:** Haopeng Xu, Yi Xia, Juan Qin, Jie Xu, Chongyan Li, Yan Wang

**Affiliations:** 1 State Key Laboratory of Ultrasound in Medicine and Engineering, College of Biomedical Engineering, Chongqing Key Laboratory of Biomedical Engineering, Chongqing Medical University, 400016 Chongqing, China; 2 Department of Gynaecology, Guiyang Maternal and Child Health Hospital, Guizhou, 550003 China

**Keywords:** POF, LIPUS, VCD, Apoptosis, Bax and Bcl-2

## Abstract

**Background:**

Premature ovarian failure (POF) is a common disease in the field of Gynecology. Low intensity pulsed ultrasound (LIPUS) can promote tissue repair and improve function. This study was performed to determine the effects of LIPUS on granulosa cells (GCs) apoptosis and protein expression of B-cell lymphoma-2 (Bcl-2) and BCL2-Associated X (Bax) in 4-vinylcyclohexene diepoxide (VCD)-induced POF mice and investigate the mechanisms of LIPUS on ovarian function and reserve capacity.

**Methods:**

The current POF mice model was administrated with VCD (160 mg/kg) by intraperitoneal injection for 15 consecutive days. The mice were divided into the POF group, LIPUS group and control group. In the LIPUS group, the right ovary of mice was treated by LIPUS (acoustic intensity was 200 mW/cm^2^, frequency was 0.3 MHz, and duty cycle was 20%) for 20 min, 15 consecutive days from day 16. The mice of the POF group and control group were treated without ultrasonic output. The basic observation and body weight were recorded. Hematoxylin and eosin staining (H&E staining) and enzyme-linked immunosorbent assay (ELISA) were applied to detect ovarian follicle development, ovarian morphology and sex hormone secretion. Ovarian GCs apoptosis was detected by TUNEL assay and immunohistochemistry.

**Results:**

The results showed that VCD can induce estrus cycle disorder, follicular atresia, sex hormone secretion decreased and GCs apoptosis in mice to establish POF model successfully. LIPUS significantly promoted follicular development, increased sex hormone secretion, inhibited excessive follicular atresia and GCs apoptosis. The mechanism might be achieved by increasing the protein expression of Bcl-2 and decreasing the expression of Bax in ovaries.

**Conclusions:**

LIPUS can improve the POF induced by VCD. These findings have the potential to provide novel methodological foundation for the future research, which help treat POF patients in the clinic.

## Introduction

Premature ovarian failure (POF), a common disease in the field of Gynecology, is endocrinology pathologically characterized by amenorrhea, infertility, estrogen deficiency, and increased gonadotropin levels in women [1, 2]. The incidence of POF is 1% before the age of 40, which induces multiple health risks, including night sweats, insomnia, mood changes, blood pressure fluctuations, a series of syndromes of osteoporosis and cardiovascular disease [3, 4]. Therefore, modeling and treatment of POF have been paid more attention in recent years.

According to different pathological manifestations, there are many approaches to establish POF model in an animal. The most common is irreversible pathogenesis of POF caused by chemotherapy drugs such as cyclophosphamide and busulfan, which could induce follicle loss, vascular damage and tissue fibrosis [5–7]. But the chemotherapy drugs damage other tissues and organs in addition to ovarian tissue and increase the risk of death during modeling [8]. Besides, iatrogenic surgery and gene knockout were also successful in establishing animal models of POF. However, these two methods have the disadvantage of simulating only part of POF and high costs respectively [9, 10]. Our present study was performed on the POF mice model induced by 4-vinylcyclohexene diepoxide (VCD). VCD is a novel ovarian toxic substance that induces POF to exhibit unique advantages in dynamic simulation of human ovarian decline in an animal model [11]. The VCD-induced POF model has a high success rate and the persistent effects [12]. Apoptosis of oocyte and granulosa cells (GCs) has been identified as one possible underlying mechanism for VCD-induced POF, which leads primordial and primary follicles to be destroyed [13, 14]. B-cell lymphoma-2 (Bcl-2) and BCL2-Associated X (Bax) are regulators of primordial follicle initiation and apoptosis, and the ratio of Bcl-2 /Bax determines whether cell apoptosis occurs when stimulated by apoptosis signal [15, 16].

At the same time, the prevention and treatment of POF are particularly difficult. Hormone replacement therapy (HRT) is considered as the most commonly used treatment at home and abroad, but it cannot really restore the endocrine function of the ovaries [17]. Recently, it’s reported that in vitro activation (IVA) of dormant follicles a potential and effective approach for infertility treatment of patients with primary ovarian insufficiency (POI) [18]. Low intensity pulsed ultrasound (LIPUS) is defined as a safe and effective therapy that has been studied in recent years and used to help treat many diseases [19]. As an emerging physical therapy, LIPUS can promote bone healing, soft-tissue regeneration, inhibition of inflammation and other applications [20]. It has also been found that LIPUS could help promote the recovery of ovarian histological structure in rats with ovarian injury induced by cyclophosphamide [21].

Starting from the modeling and treatment of POF, we performed the effects of LIPUS in POF mice induced by VCD in this study. The aim of our study is to investigate ovarian function and reserve capacity, GCs apoptosis and related Bcl-2 and Bax. The results might introduce a new method to help treat POF in clinics in the future.

## Materials and methods

### Animal

A total number of 36 female C57BL/6 mice (weight 20 ± 2 g and age 6–8 weeks) used in this study were obtained from the Experimental Animal Center of Chongqing Medical University (production license number: SCXK 2018–0003). All experimental animals were maintained under standard conditions (humidity 45% ± 5%, temperature 23 ± 5 °C, light time 12 h/d). The animal experimental protocol was according to the ethical standards of the Experimental Animal Ethics Committee of Chongqing Medical University. Animal handling and experimental procedures were performed in accordance with the Guide for the Care and Use of Laboratory Animals issued by the Ministry of Science and Technology, China. All animal experiments were conducted at the Experimental Animal Center, Chongqing Medical University, China.

The mental state of the mice, weight, diarrhea, hair color and activity were observed and recorded during the experiment.

### POF model establishment and grouping

The mice were allowed to acclimatize for 1 week. Then, they were randomly divided into the three following groups with 12 mice per group: LIPUS group (n = 12), POF group (n = 12) and control group (n = 12). The VCD-induced POF mice model was established according to the literature [22]. The mice in the POF group and LIPUS group were intraperitoneally injected daily with VCD (Shanghai Aladdin Biochemical Technology Co. LTD, Shanghai, China; 160 mg.kg^−1^. d^−1^) for 15 days, the mice in the control group received an equal volume of saline (i.p. daily) for 15 days.

### Protocol of LIPUS treatment

The LIPUS device (Chongqing Haifu Medical Technology Co. LTD, Chongqing, China) was used in this study. The acoustic intensity was 200 mW/cm^2^ measured by a sound power meter (UPM-DT-1AV, Ohmic Instruments Co., St. Charles, MO, USA) in degassed water. The frequency of the transducer was 0.3 MHz and the duty cycle was 20%. Before LIPUS treatment, the skin of the lower abdomen and the right ovary was depilated, disinfected, and covered with the ultrasonic coupling agent (Tianjin Chengxin Medical Auxiliary Material Factory, Tianjin, China). Then, LIPUS started to work. The mice of the LIPUS group received ultrasound treatment for 20 min per day for 15 consecutive days after successful POF mice modeling. The control group and the POF group were handled as the same as the LIPUS group except for no energy output from LIPUS device.

### Estrus cycle examination

The mice were examined with a vaginal smear of exfoliated cells by fine cotton swabs during a 15-day period following VCD injection. After natural drying, the slides were fixed in 95% alcohol solution (Chongqing Chuandong Chemical Co. LTD, Chongqing, China) for 10 min and stained in 0.23% alkaline methylene blue (Beijing Solarbio Technology Co. LTD, Beijing, China) for 5 min. Vaginal smear was performed under an optical microscope (BX51, Olympus, Tokyo, Japan). The type of estrus cycle was determined as shown by the proportions of nucleated and keratinized epithelial cells and leukocytes. Estrus cycle disorder is a distinguishing characteristic of ovarian function failure [23].

### Hematoxylin and Eosin (H&E) staining

After 15 days of LIPUS treatment, the mice were over-anesthetized to be euthanized and their right ovaries were taken out completely. Sections were observed by H&E staining to analyze the ovarian morphology and follicle counts. The ovarian tissues were collected from each group and fixed with 4% paraformaldehyde (Biosharp Co. LTD, Anhui, China) for 48 h. After H&E staining, sections were observed under an optical microscope. Follicles were counted and classified as primordial, primary, secondary, preovulatory and atretic follicles, respectively [24]. Five nonrepetitive views on each slide were selected for statistical analysis.

### Enzyme-linked immunosorbent assay (ELISA)

For detection of sex hormones (estradiol (E2) and Anti-Mullerian hormone (AMH)), blood samples were obtained from eyeball veins of mice in each group (n = 8) and centrifuged at 3000 rpm for 15 min at the last day of LIPUS treatment. Serum levels of sex hormones was determined by ELISA kits (Jiangsu Jingmei Biological Technology Co., Ltd, Jiangsu, China) following the manufacturer’s instructions.

### TUNEL assay

Ovarian GCs apoptosis was detected by the TUNEL apoptosis assay kit (4A Biotech Co. LTD, Beijing, China) according to the manufacturer’s instructions. The fluorescence was observed under a fluorescence microscope (IX73, Olympus, Tokyo, Japan). Ovarian apoptotic cells were stained bright red. Five fields were randomly selected from sections, and the apoptotic index (AI) was calculated by Image J (National Institutes of Health, USA).

### Immunohistochemistry

The slides were dewaxed and antigen repaired, then incubated with the primary polyclonal rabbit antibodies of Bax and Bcl-2 (Affinity Biosciences LTD, OH. USA). The concentration of Bax and Bcl-2 was 1:150 and antibodies were incubated for 2 h at 37 °C in a water bath pot (Wiggens Co. LTD, Germany). Biotinylated secondary antibody anti-rabbit IgG (Fuzhou Maixin Biotechnology Development Co. LTD, Fuzhou, China) was used on the sections for 40-min incubation at indoor temperature. Then the slides were colored with DAB (Beijing Zhongshan Jinqiao Biotechnology Co. LTD, Beijing, China). Five sections on each slide were selected randomly for examination under an optical microscope and the Image J was used for quantitative analysis.

**Fig. 1:**
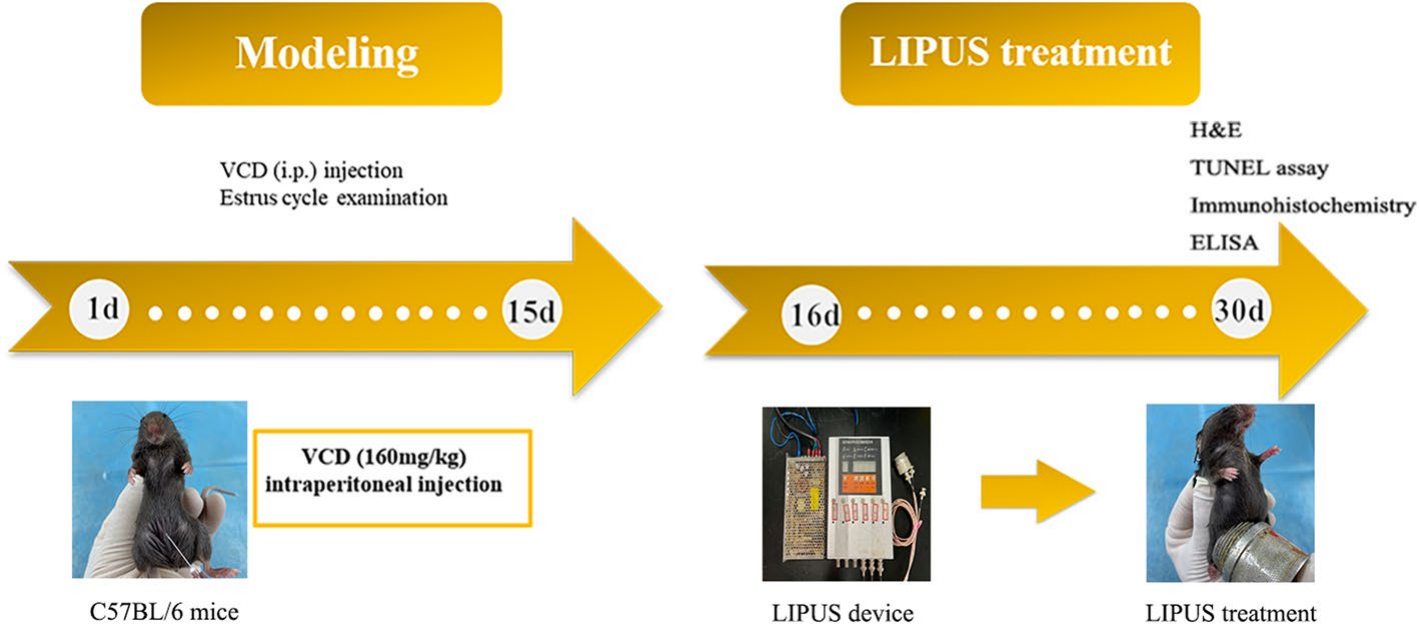
A schematic diagram of research plan

A schematic diagram of research plan could be seen in Fig. 1.

### Statistical analysis

All the data was expressed as mean ± standard deviation. Data were statistically analyzed using the SPSS 22.0 software (IBM, Armonk, New York, USA). Independent-samples t-test was used to analyze the difference in groups. A *p*-value < 0.05 was considered statistically significant.

## Results

### The general situation of mice

To investigate the general situation of mice, the mice were observed from modeling to treatment. None of the mice had diarrhea during this period. After (i.p.) injection with VCD, the POF mice were depressed, had no glossy hair and poor activity. Compared with the POF group, the mice in the LIPUS group showed glossy hair and increased activity. In the control group, the mice had glossy hair, good activity, a normal diet, and a good response to external stimuli.

### Body and reproductive organ weights

**Fig. 2:**
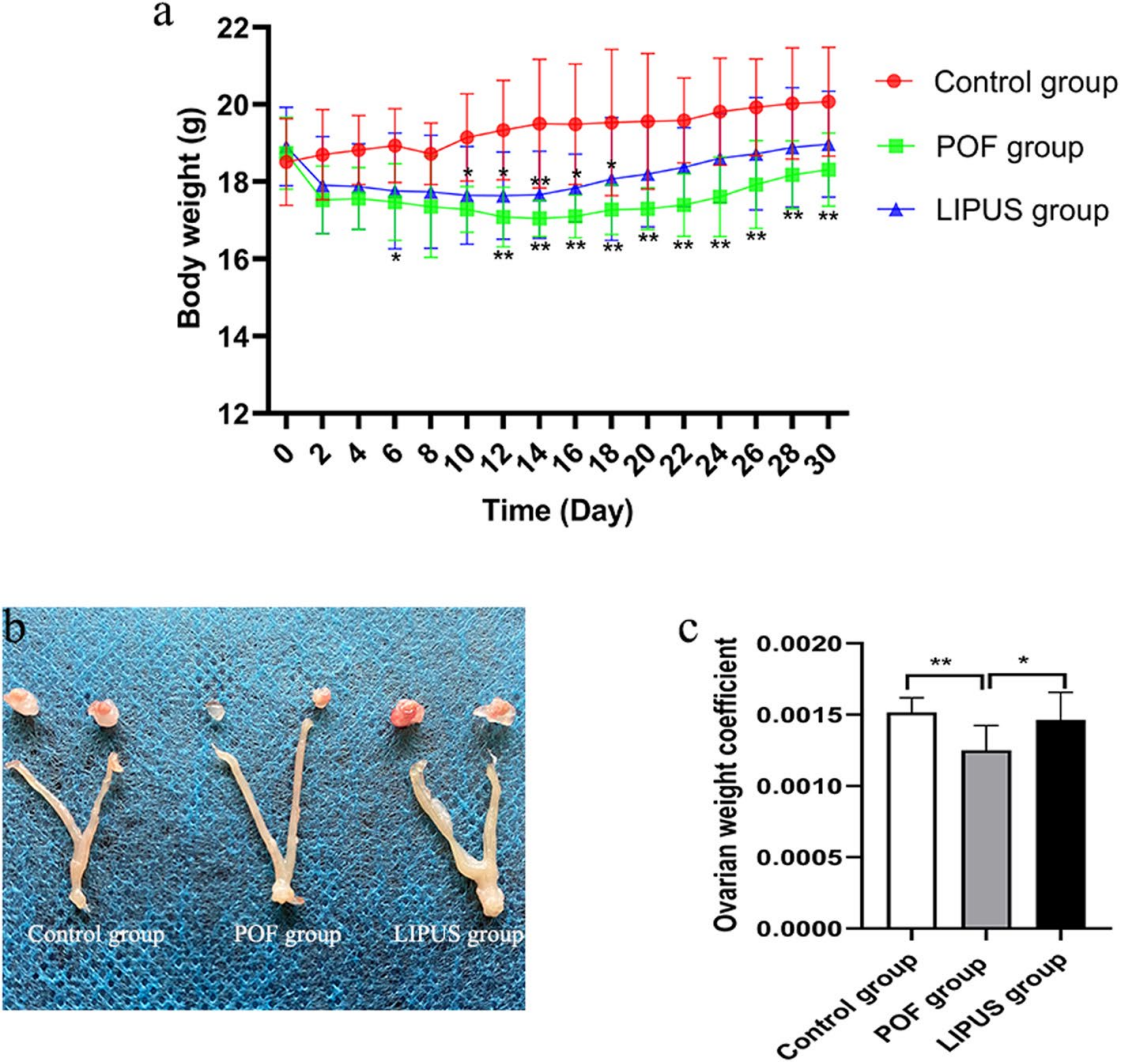
Body weights and reproductive organ weights of the mice. **a** The changes in body weight before and after LIPUS treatment. **b** Representative photographs of ovaries and uteruses. **c** Ovarian weight coefficient. *p < 0.05, **p < 0.01

The body and reproductive organ weights of the mice were investigated next. Our results showed that the body weights of mice were significantly decreased (Fig. 2a) after VCD injection. Moreover, compared to the POF group, the body weights in LIPUS group were significantly increased, starting from the LIPUS treatment. As shown in the Fig. 2b-c, the weight coefficient of right ovary in the POF group was significantly decreased (*p* < 0.05), while the weight coefficient of ovary was significantly increased after LIPUS treatment. These results demonstrated that LIPUS can restore the body and reproductive organ weights of POF mice.

### Effects of VCD on estrus cycles

**Fig. 3:**
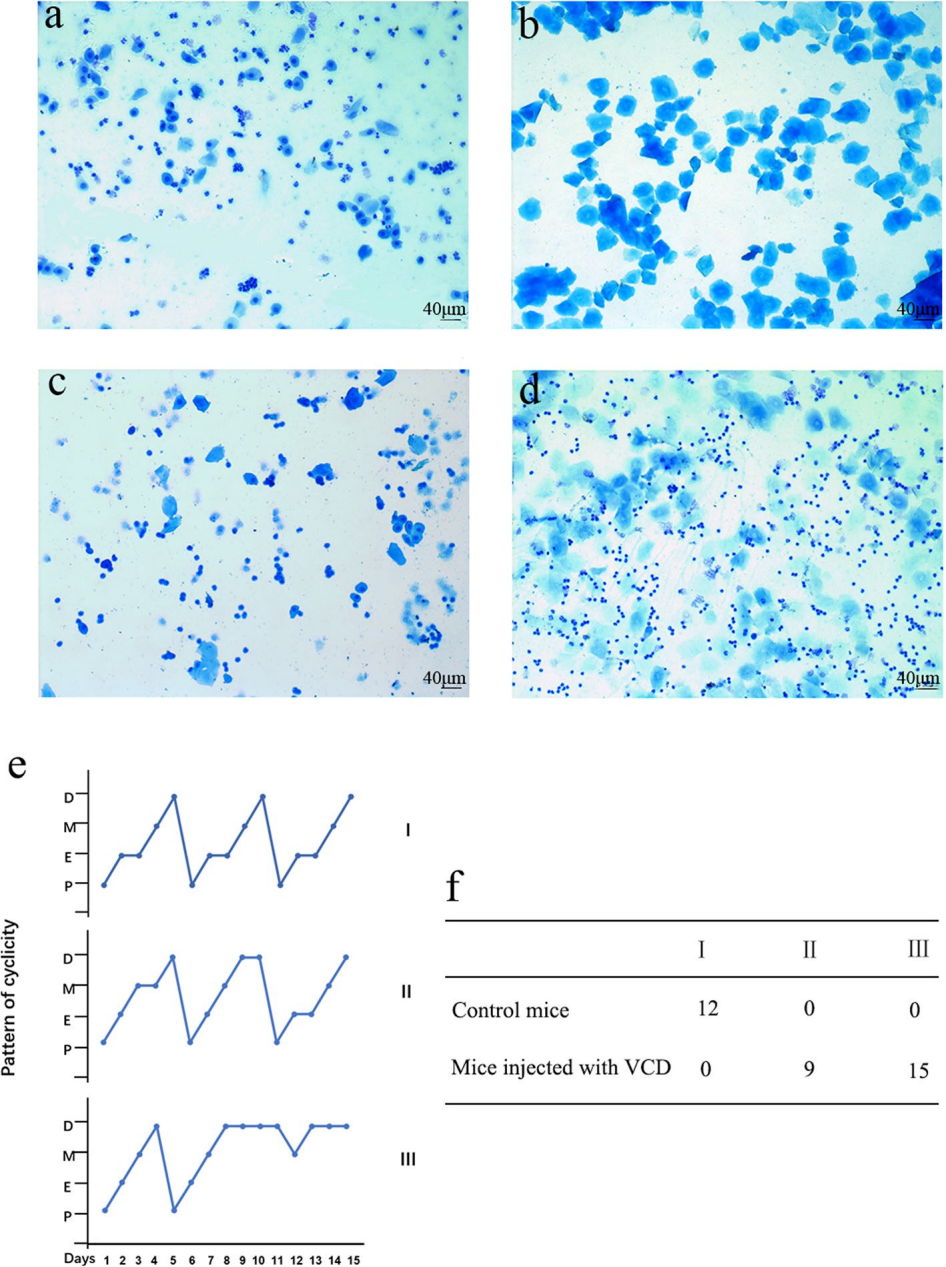
Effect of 4-vinylcyclohexene diepoxide (VCD) on estrus cycles in mice. Vaginal smears were obtained and the estrus cycles were evaluated by 0.23% alkaline methylene blue staining. Representative photographs: **a** proestrus, **b** estrus, **c** metestrus, **d** diestrus (100 ×). **e** Three patterns of estrous cycles were graded with the severity of abnormality (I–III) as follows: I, normal II, irregular cycles with a prolonged diestrus and normal or prolonged estrus III, no cyclicity. Bars represent the average length (day) of proestrus (P), estrus (E), metestrus (M) and diestrus (D) per estrous cycle. f Total numbers of mice from each group categorized into the various estrous patterns (I–III)

In order to better verify the establishment of POF mice model, the estrus cycles were examined during a 15-day period following VCD injection. The type of estrus cycle was determined as shown by the proportions of nucleated and keratinized epithelial cells and leukocytes (Fig. 3a-d). Regular estrus cycles in the control group were shown in all mice and lasted 4–5 days, which includes proestrus for 1 day, estrus for 1–2 days, metestrus for 1 day and diestrus for 1–2 days. However, the significantly irregular estrus cycles were observed after injection with VCD as shown in Fig. 3e. There were irregular estrus cycles with a prolonged diestrus and normal or prolonged estrus in 9 mice, and the other 15 had no cyclicity (Fig. 3f). The results indicated that the VCD-induced POF mice model was established successfully.

### Histological changes and follicle count

**Fig. 4:**
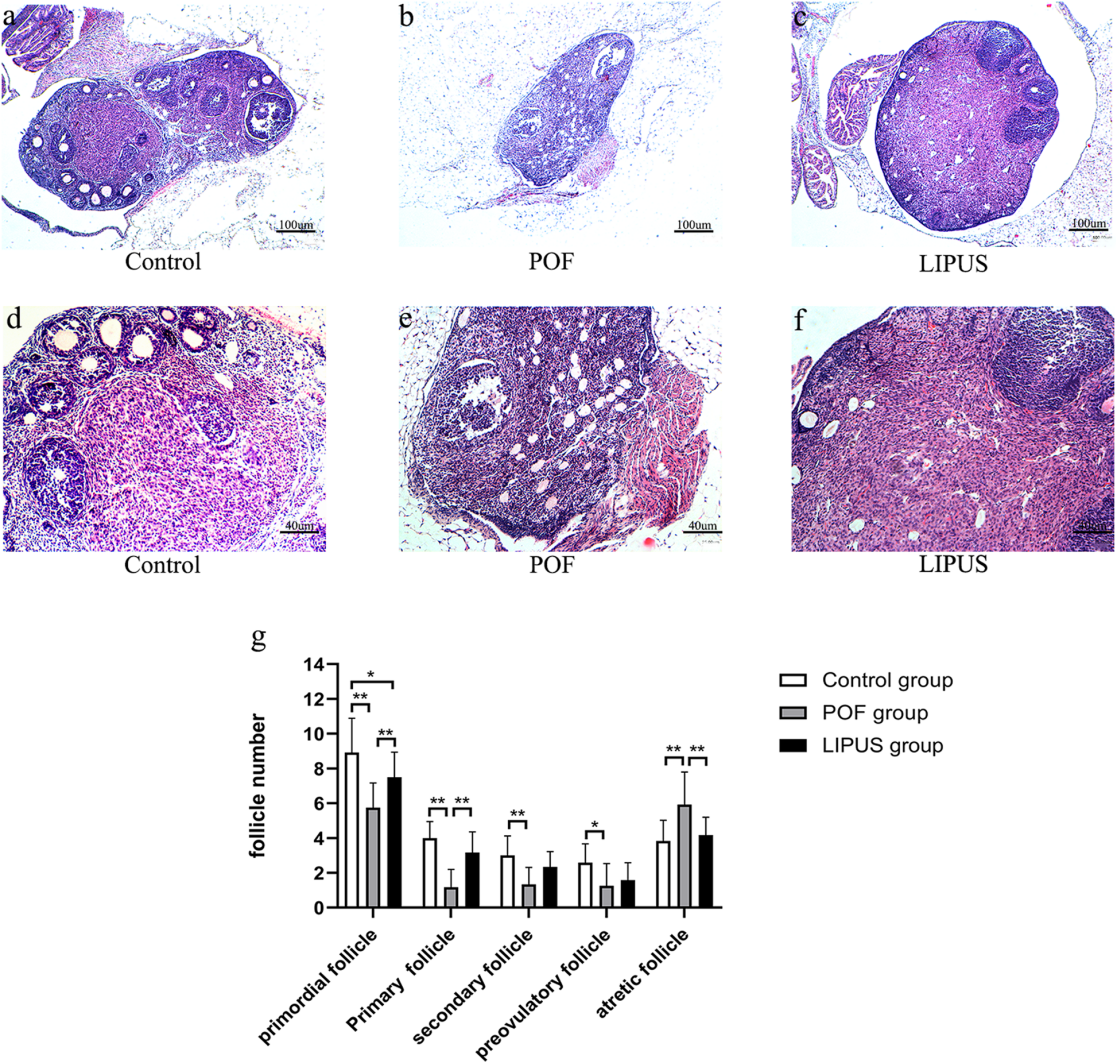
Effect of low intensity pulsed ultrasound (LIPUS) on follicle growth and ovary in (premature ovarian failure) POF mice. **a**-**f** the pathological changes of ovaries (**a**-**c**: 40 ×) and follicles (**d-f**: 100 ×) were evaluated by H&E staining in the three groups. **g** the number of follicles at various stages were counted and compared in the three groups. *p < 0.05, **p < 0.01

Histologic changes and follicular count reflect the reserve capacity of the ovary. The right ovary in the control group was of normal size, with multiple growth follicles at different stages in the cortex, and the follicle fluid was deeply stained. In the POF group, the ovarian atrophied formlessly and the number of follicles decreased significantly, of which most were atresia follicles with less follicular fluid. Compared with the POF group, the ovarian volume of the LIPUS group was larger, and follicles of various stages were visible (Fig. 4a-f). As shown in Fig. 4g, compared with the control group, the number of primordial and primary follicles in the POF group was significantly reduced, the number of atresia follicles was significantly increased (*p* < 0.05), and the number of follicles in other stages decreased (*p* < 0.05). Compared with the POF group, the number of atresia follicles in the LIPUS group was significantly reduced (*p* < 0.05), while the number of primordial follicles, primary follicles and secondary follicles was significantly increased (*p* < 0.05). It was found that VCD could kill small follicles including primordial and primary follicles, while LIPUS could help improve ovarian reserve capacity.

### The serum of E2 and AMH levels

**Fig. 5:**
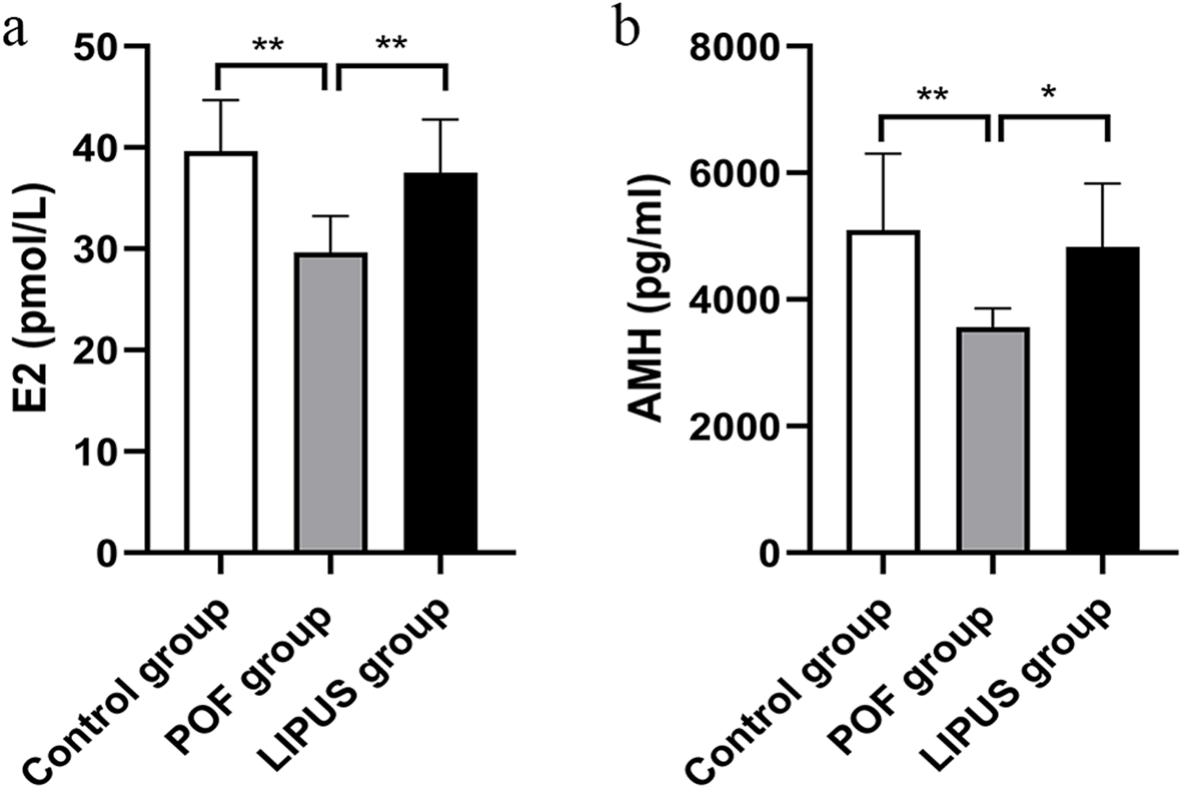
ELISA analysis of sex hormones. At the last day of LIPUS treatment (day 30), serum was collected for ELISA analysis of E2 and AMH. E2: estradiol; AMH: Anti-Mullerian hormone. *p < 0.05, **p < 0.01

Changes in sex hormones such as E2 and AMH are pathologic features of POF. As shown in Fig. 5, the serum of E2 and AMH levels in the POF group were lower than those in the control group (*p* < 0.05). Compared with the POF group, the serum of E2 and AMH levels were increased (*p* < 0.05) in the LIPUS group. The results showed that VCD could lower the serum of E2 and AMH, while LIPUS raised them in the POF mice.

### Granulosa cell apoptosis

**Fig. 6:**
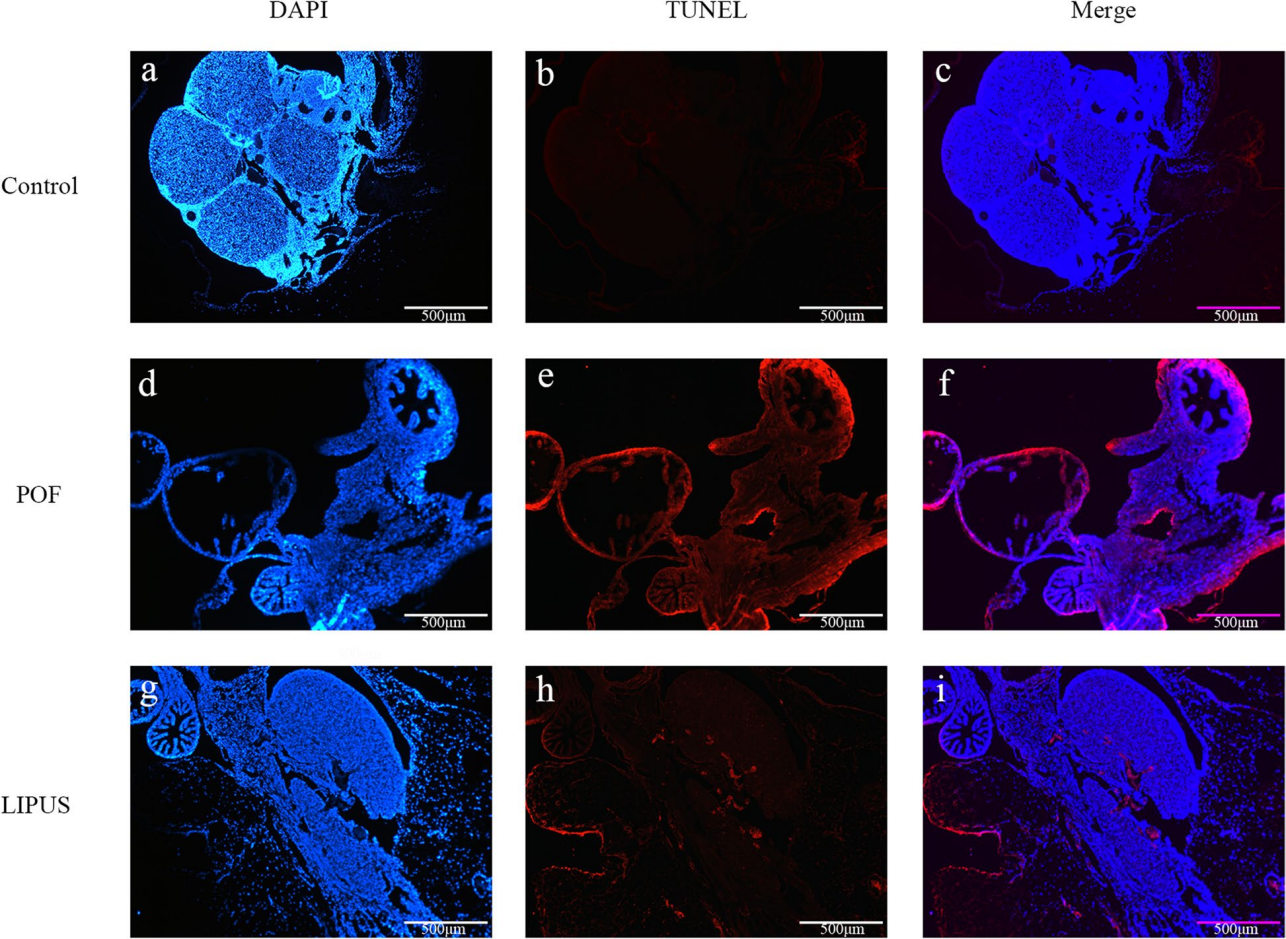
Apoptosis of granulosa cells (GCs) in ovarian tissues were measured by TUNEL assay. **a, d, g** the nucleus was stained with DAPI, shown as blue fluorescence (40 ×). **b, e, h** Apoptotic cells shown as red fluorescence with TUNEL assay (40 ×). **c, f, i** Figures were merged by Image J (40 ×)

**Fig. 7:**
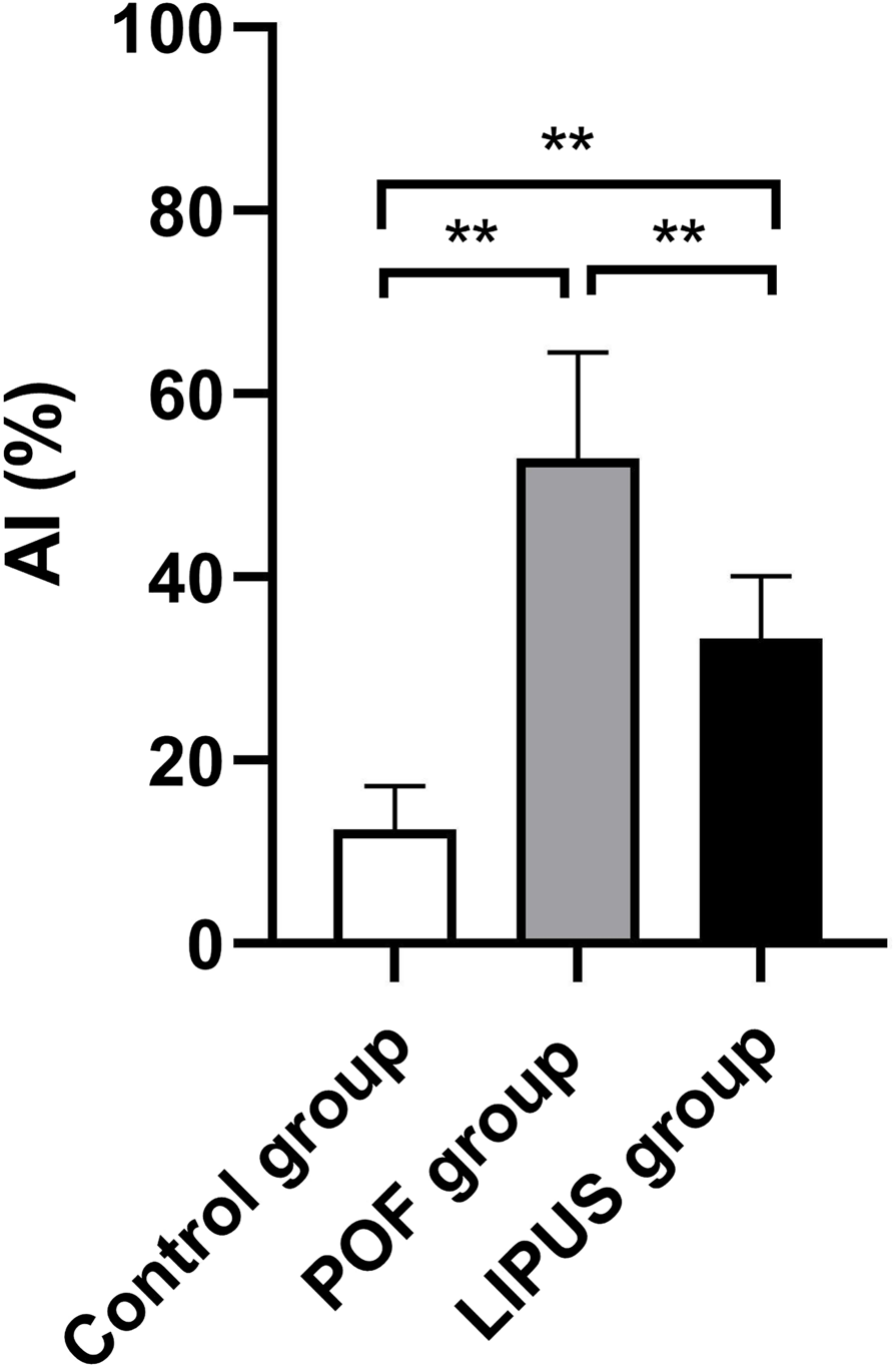
The apoptosis index (AI) of three groups. *p < 0.05, **p < 0.01

Apoptosis of GCs is closely related to the development of follicular atresia. The results revealed that the number of apoptotic cells in the POF group was significantly increased compared to the control group (Fig. 6). However, after LIPUS treatment, the apoptosis numbers of GCs were significantly decreased. As shown in the Fig. 7, the data obtained by Image J analysis showed that the AI of LIPUS group was lower than that in the POF group (*p* < 0.05). The results showed that LIPUS prohibited the GCs from apoptosis in POF mice.

### Expression of Bax and Bcl-2

**Fig. 8:**
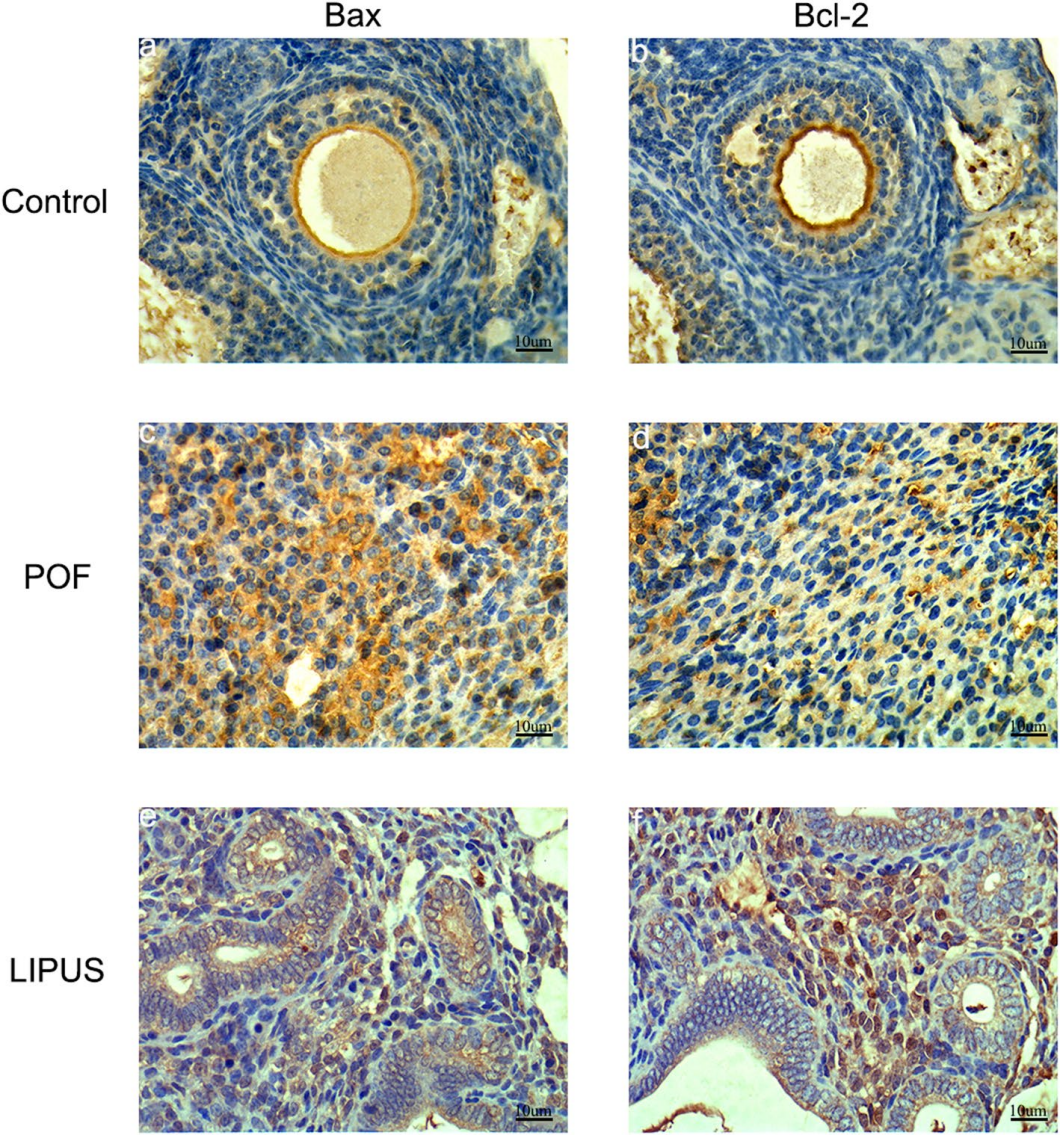
Effect of low intensity pulsed ultrasound (LIPUS) on the protein of Bax and Bcl-2 in premature ovarian failure (POF) mice. Protein expression of Bax and Bcl-2 was evaluated by immunohistochemistry. **a-b** control group **c-d** POF group **e–f** LIPUS group (400 ×)

To further study the mechanism of GCs apoptosis and the follicular atresia, protein expression of Bax and Bcl-2 was detected by immunohistochemical analysis. As shown in Fig. 8, Bax and Bcl-2 proteins were expressed in the follicles of mice in each group, and the positive region showed brown-yellow reaction particles. The data obtained by Image J analysis (Table 1) showed that the expression of Bcl-2 was decreased and Bax was increased in ovaries of VCD -induced POF mice compared to the control group (*p* < 0.05). After LIPUS treatment, compared with the POF group, the expression of Bcl-2 increased and Bax decreased (*p* < 0.05). Furthermore, the ratio of Bcl-2 /Bax was significantly increased in the LIPUS group than that in the POF group (*p* < 0.05). This finding indicated that LIPUS could improve the apoptosis of GCs by increasing the ratio of Bcl-2 /Bax.

**Table 1:** Expression of Bax, Bcl-2 and Bcl-2/Bax in three groups (‾x ± s)

Group	n	Bax (AOD)	Bcl-2 (AOD)	Bcl-2/Bax
Control	12	0.134 ± 0.014**	0.207 ± 0.044**	1.495 ± 0.145**
POF	12	0.207 ± 0.036▲▲	0.139 ± 0.022▲▲	0.646 ± 0.052▲▲
LIPUS	12	0.173 ± 0.026*▲▲	0.173 ± 0.023**▲	1.001 ± 0.069**▲▲

Compared with the control group, ▲*p* < 0.05, ▲▲*p* < 0.01, Compared with the POF group, *p < 0.05, * *p < 0.01

## Discussion

Approximately 10% of worldwide women of age between 30 and 39 are suffering from POF [25]. A variety of factors have been found to be associated with POF including genetic defects, infections, autoimmunity, drugs, and toxics [26]. In our study, the POF mice model was established by VCD. VCD is an ototoxic catabolite of the occupational chemical 4-vinylcyclohexene, an intermediate used in the manufacture of flame retardants, flavors, pesticides, adhesives, fragrances, and synthetic rubber [27, 28]. VCD has long been considered a hazardous occupational chemical that promotes POF and it can well simulate the dynamic process of human POF [11]. Prior studies have consistently indicated that VCD induces atretic degeneration of primordial and primary follicles in rodents and primates [13, 29]. The results of our study also demonstrated that compared with the control group, the ovarian volume shrank, the primordial and primary follicles decreased (*p* < 0.05), and the atretic follicles increased significantly (*p* < 0.05) in the POF group. On the other hand, estrus cycle disorder is a distinguishing characteristic of ovarian function failure, which was consistent with our results. It has been seen that VCD-induced POF model is safe, has a high success rate, and is relatively stable.

The ovary with periodic development is one of the few organs that fully reflect cell apoptosis. The regulation of follicular development was mainly affected by the local cytokines of the ovary, and the interactions between the GCs and the oocytes played an important role in regulation [30–32]. It’s reported that the follicles become atretic when 10% of GCs have undergone apoptosis [33]. This indicates that GC apoptosis is of great importance in the development of follicular atresia. Regulation of cell apoptosis is a very complex process. Ovarian GC apoptosis follows two main pathways: death receptor and mitochondrial pathways [34]. In the earlier pathway, the specific death ligands will combine with death receptors, thus activating caspase cascade [35]. In the mitochondrial pathway, it is mainly regulated and activated by the Bcl-2 family, among which pro-apoptotic molecules and anti-apoptotic molecules (Bax and Bcl-2) are the most common apoptotic markers, and Bax will initiate the apoptosis and promote follicular atresia [15, 36]. In the process of cell apoptosis, Bax moves to mitochondria from cytoplasm, which subsequently enhances the mitochondrial membrane permeability. BCL-2 prohibits the cytochrome C elimination from entering into the cytoplasm, thus acting antagonistically on BAX and opposing cell apoptosis [37, 38]. Therefore, Bcl-2/Bax acts as a regulator in follicular maturation and atresia. The ratio of Bcl-2 /Bax determines whether cell apoptosis occurs when stimulated by apoptosis signal, which determines the fate of ovarian GCs [16, 39]. Consistent with previous reports, in this study, we demonstrated that compared with the control group, the mice ovarian GC AI index in the POF group was significantly increased (*p* < 0.05), Bax and Bcl-2 formed homologous dimers, and the ratio of Bcl-2 /Bax decreased (*p* < 0.05), which promoted the apoptosis of ovarian GCs.

In recent years, LIPUS has become a new and non-invasive treatment method. LIPUS is a form of mechanical vibration energy transmission [40]. The acoustic pressure wave produced by LIPUS is able to transmit into and through living cells, which may result in a series of biochemical events at the cellular level [40, 41]. In the early 1970s, it was found that small doses of ultrasound boosted the formation of follicles in the ovaries of mice [42]. The characteristics of ovarian dynamics are related to the repeated proliferation and differentiation of follicular cells [43]. Recent study has also shown that LIPUS has a certain effect on the proliferation of mesenchymal stem cells derived from human ovarian follicular fluid [44]. Lin et al. found that LIPUS-pretreated human amnion-derived mesenchymal stem cell (hAD-MSCs) transplantation can repair ovarian injury and improve ovarian function in rats with chemotherapy-induced POI, which may be attributed to the increased growth factors in hAD-MSCs promoted by LIPUS [45]. In our study, LIPUS also helps improve the VCD-induced POF mice ovarian function, reduce ovarian GC apoptosis and restore follicular development. HE staining showed that the primordial and primary follicles of POF mice increased and the atresia follicles decreased after LIPUS treatment. At the same time, apoptosis of granulosa cells plays an important role in the development of ovarian function failure in POF mice. LIPUS could inhibit ovarian GCs apoptosis to recover ovarian function. Furthermore, the ratio of Bcl-2 /Bax was increased by immunohistochemistry. It’s suggested that LIPUS can affect mitochondrial pathways regulated by the Bcl-2 family to oppose GC apoptosis. Besides, Recent studies indicate involvement of the Hippo signaling pathway in the regulation of follicle growth [46]. This signaling pathway is also known to regulate cell proliferation and apoptosis to maintain normal organ size [47]. Our results suggest that the effect of LIPUS on POF mice may be related to this signaling pathway, particularly in the regulation of follicular growth. We need further studies to prove the connection between LIPUS and Hippo signaling pathway.

There are two main limitations to our study. First, it is necessary to further explore whether LIPUS also affects the left of ovary in mice, and there is also a need to increase the duration of ultrasound intervention. In addition, the increased Bcl-2 /Bax ratio may be related to the promotion of intra-ovarian growth factors by LIPUS, which needs to be confirmed by further studies.

## Conclusion

In conclusion, LIPUS can obviously promote follicular development, inhibit excessive follicular atresia and granular cell apoptosis, and improve the ovarian reserve capacity in POF mice. We found that the expression of anti-apoptotic protein Bcl-2 was elevated, and apoptotic protein Bax was decreased, which prohibits the GCs from apoptosis via mitochondrial pathway in the POF mice. It has the potential to provide a promising therapeutic method for patients with POF in the clinic.

## Data Availability

The datasets used and/or analysed during the current study are available from the corresponding author on reasonable request.

## Abbreviations

POF: Premature ovarian failure
LIPUS: Low intensity pulsed ultrasound
GCs: Granulosa cells
VCD: 4-Vinylcyclohexene diepoxide
Bcl-2: B-cell lymphoma-2
Bax: BCL2-Associated X
HRT: Hormone replacement therapy
E2: Estradiol
AMH: Anti-Mullerian hormone
AI: Apoptotic index
